# Bone-like ceramic scaffolds designed with bioinspired porosity induce a different stem cell response

**DOI:** 10.1007/s10856-020-06486-3

**Published:** 2021-01-20

**Authors:** Silvia Panseri, Monica Montesi, Dominique Hautcoeur, Samuele M. Dozio, Shaan Chamary, Eamonn De Barra, Anna Tampieri, Anne Leriche

**Affiliations:** 1 Institute of Science and Technology for Ceramics, National Research Council, Faenza, Italy; 2 Belgian Ceramic Research Centre, Avenue Gouverneur Cornez 4, B-7000 Mons, Belgium; 3 Université Polytechnique Hauts-de-France, Laboratoire des Matériaux Céramiques et Procédés Associés, 59313 Valenciennes, France; 4 University of Limerick, Bernal Institute, Limerick, V94 T9PX Ireland

## Abstract

Biomaterial science increasingly seeks more biomimetic scaffolds that functionally augment the native bone tissue. In this paper, a new concept of a structural scaffold design is presented where the physiological multi-scale architecture is fully incorporated in a single-scaffold solution. Hydroxyapatite (HA) and β-tricalcium phosphate (β-TCP) bioceramic scaffolds with different bioinspired porosity, mimicking the spongy and cortical bone tissue, were studied. In vitro experiments, looking at the mesenchymal stem cells behaviour, were conducted in a perfusion bioreactor that mimics the physiological conditions in terms of interstitial fluid flow and associated induced shear stress. All the biomaterials enhanced cell adhesion and cell viability. Cortical bone scaffolds, with an aligned architecture, induced an overexpression of several late stage genes involved in the process of osteogenic differentiation compared to the spongy bone scaffolds. This study reveals the exciting prospect of bioinspired porous designed ceramic scaffolds that combines both cortical and cancellous bone in a single ceramic bone graft. It is prospected that dual core shell scaffold could significantly modulate osteogenic processes, once implanted in patients, rapidly forming mature bone tissue at the tissue interface, followed by subsequent bone maturation in the inner spongy structure.

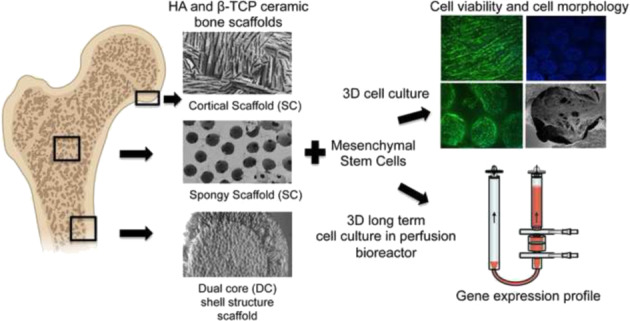

## Introduction

Human bone includes a heterogeneous combination of strong, stiff cortical bone with a functional, low modulus cancellous core [1]. Although it has a certain capability for regeneration and self-repair, large segmental bone defects caused by trauma, cancer surgical removal, or congenital disorders can only be repaired by bone grafting [2, 3]. In bone scaffold design, several features are fundamental in influencing the biological response and guide the tissue regeneration: (i) implant raw material; (ii) surface chemistry; (iii) size, shape and porosity; (iv) biodegradability [4–6]. The main goal is to mimic the extra cellular matrix microenvironment achieving the complex cell/material interactions able to trigger and support the regeneration process [7, 8]. Within the bone biomaterial world, bioceramics play a pivotal role in tissue regeneration due to their chemical mimesis of the inorganic component of bone tissue. As the most representative bioactive ceramics, Ca–P ceramics, including hydroxyapatite [HA, Ca_10_(PO_4_)_6_(OH)_2_] and β-tricalcium phosphate [β-TCP, Ca_3_(PO_4_)_2_], have compositions similar to the natural bone [9–11]. HA and β-TCP are biocompatible and bioactive. Once implanted in vivo, the multiple interactions between material and host provide local adequate conditions for cell attachment, proliferation and differentiation [10, 12–14].

Degradation products (i.e., released ions) can enter in cell metabolism and create an alkaline and calcium rich *milieu* to boost cell activity and make the tissue regeneration process faster [3]. The major difference between HA and β-TCP is related to the degradation rate in vivo. In fact β-TCP is more degradable and becomes soluble more rapidly; its degradation rate is 10–20 times higher than that of HA [15]. Although enormous advancements have been made in recent decades, large bone defect regeneration remains a challenge in medicine [16, 17]. There are a number of successful Ca-P ceramic scaffolds or fillers already available on the market used in cancellous bone replacement in non load-bearing indications (JectOS/AREX®BONE, CustomBone®, CronOS®, Engipore®). Unfortunately clinical solutions for cortical bone regeneration remain extremely limited [9, 18–22].

Although many studies have focussed on developing “the perfect biomaterial” with a combination of suitable physico-chemical, mechanical and biological properties, the complete regeneration of critical bone defects remains a great challenge in medicine. Natural bone, in fact, shows a multi-scale structure that confers unique features on the type and location of the tissue. Broadly, bone can be divided into two types: cancellous, distributed inside the bone, and cortical bone located at the surface on the bone. Several techniques have been employed in bone scaffold design in order to reach the closest biomimesis of this hard tissue. Unfortunately so far there are no commercially available bone grafts that recapitulate both structures [23–25] in a single construct. In addition, to be successful, a bone regeneration strategy must consider the bioactivity of the bone graft itself as well as its topography and macrostructure, as the chemistry plays a fundamental role in modulating cell behaviour and orchestrating the complex regenerative process [26–28]. Of all the biomaterials, CaP based bioceramics still represent the best biomimetic materials reproducing the chemistry of the organic bone phase and able to communicate to the cells accurate signals promoting bone regeneration [29–31].

The present work can be characterised as a new concept design where the physiological multi-scale structure is fully included in one-scaffold solution, opening new horizons in complex architecture bone scaffold materials. Cortical bone plays a fundamental role in bone biomechanics, so its morphological features can usefully be considered and inform the strategy for regeneration of critical bone defects [1, 32]. In this study, we analysed the stem cell behaviour in response to different porosity morphologies and distributions in both HA and β-TCP ceramic bone scaffolds that mimic in detail both the spongy and cortical bone tissue architecture. The spongy-like scaffold was prepared by replica technique from polymer beads, and the cortical-like scaffold via freeze casting [33]. A perfusion bioreactor was used as predictive tool to better replicate the physiological human body process and it allows long term cell culture in vitro. Moreover, a preliminary biological evaluation was performed on a dual structure scaffold that recapitulates for the first time both cortical and cancellous bone in a single ceramic bone graft.

## Materials and methods

### Hydroxyapatite and β-TCP powder manufacturing

HA and β-TCP powders were synthesized through aqueous precipitation [34, 35]. A di-ammonium phosphate solution (NH_4_)_2_HPO_4_ (Carlo Erba, France) is added in a controlled manner to a calcium nitrate solution Ca(NO_3_)_2_ (Brenntag, France), in a jacket reactor, under mechanical stirring. Synthesis conditions such as precursors concentrations [36], pH, temperature and maturing time varied as necessary to yield the desired final product.

### Spongy scaffold (SS) manufacturing

The polymeric scaffolds were made out of partially fused polymethylmetacrylate beads (PMMA, Diakon TM Ineos Acrylics, Holland) that acted as the sacrificial phase. After being firmly packed in a mould, a solvent (acetone) was added, triggering the slow surface dissolution of the polymer, which induced fused interconnection between the individual spherical bodies. This leads to the formation of bridges between PMMA beads and a significant shrinkage of the bead pile. For a given size range of beads, interconnection/bridge diameter can be related to this displacement via a series of empirical equations and/or a geometrical model based on the theoretical arrangement of spheres [34]. In our case the size range chosen was between 500 and 600 µm with interconnections size of 150 µm. The shrinkage value was obtained by averaging results from both models. Interconnection diameter was verified through SEM imaging.

HA or β-TCP slurry was prepared with 65 wt % of powder in distilled water. Deflocculating was achieved via a commercial organic agent, Darvan C (1.5 wt. % dry matter content, R.T Vanderbilt Co., USA). Mixing took place in a milling jar for 1 h. Slurry was then transferred to a beaker under magnetic stirring for the addition of adjuvants [33]. The slurry was poured over the scaffold until its complete immersion in a plaster mould. Once dried, the scaffold was removed from the mould and the excess of material was manually removed. Before sintering, the porogens were cleanly eliminated via a slow thermal treatment: 220 °C for 20 h, followed by 250 °C for 4 h. The scaffold was densified at 1250 °C for 3 h in the case of HA SS and at 1100 °C for 3 h in the case of β-TCP SS.

### Cortical scaffold (CS) manufacturing

For the freeze casting material, slurries were prepared by mixing distilled water and HA or β-TCP powder with a small quantity (1.5 wt. % dry matter content) of ammonium polymethacrylate (Dolapix CE64, Zschimmer & Schwarz, Germany). Powder content was 68 wt. %. A small amount of binding agent, 3 wt. %, of polyethylene glycol (Mw = 1000 mol.g^−1^, Merck, Germany) was added in the suspension [37]. The slurry was poured in Teflon® mould at −20°. The freezing rate was regulated at 1 °C/min until −40 °C was reached. The temperature was maintained until complete solidification. The sample was then freeze-dried for 24 h (HETO CD8, Thermo Fisher Scientific, USA). The green sample was sintered at 1250 °C for 3 h in the case of HA CS and 1100 °C for 3 h in the case of β-TCP CS. Discs, of the correct size, are then cut off from the sintered samples.

### Dual core (DC) shell structure manufacturing

The spongy core structure was firstly prepared by the replica technique from PMMA bead scaffold impregnated with calcium phosphate slurry. The as-prepared scaffold was partially densified in order to reach its final post-sintering size after the post-sintering step. Before placing it at the centre of the freeze casting mould, a small volume of suspension was poured at −20 °C. After, more suspension was added until the complete submersion of the scaffold [33]. The same freezing procedure was followed as previously described. The association of these 2 shaping techniques, impregnation of a polymeric scaffold and freeze casting, has yielded a dual porous architecture sample presenting 2 distinct zones. The centre presents a network of spherical interconnected pores, which is the negative of the sacrificial PMMA scaffold. The outer structure exhibits the classical lamellar morphology obtained by ice templating [37]. Core and shell fractions were chosen so that each of them represented ~50% of the final volume.

### Ceramic scaffold characterization

The apparent density and porosity of the samples were determined by measuring sample mass under different conditions (dry, humid and under water) based on Archimedes’ principle using deionized water as the liquid medium. The pore size was measured by image analysis of the Scanning Electron Microscopy (SEM) micrographs (JEOL JSM 5900 LV, Japan), the average values was determined using 5 micrographs and more than 50 pores in total were investigated for each structure specimen condition using MESURIM software (ACCES, France). Three types of HA ceramic scaffolds have been tested under compression to determine the ultimate compressive strength as a function of the macrostructure. The specimens were surface grinded in order to obtain a cylinder with consistent and even surfaces for testing. Compression tests were carried out on the cylindrical samples specimens (height of 19 mm and diameter of 11 mm for SS and CS or 16 mm for DC) parallel to the freezing direction of the specimen, for cortical scaffolds and dual core shell structure, using the Instron test machine 1114 (Instron, USA) at a crosshead speed of 0.5 mm/min^−1^.

### In vitro stem cell culture

Human adipose tissue-derived stem cells (hADSCs, ATCC) were used for the biological study. In detail hADSCs were cultured in αMEM Glutamax (Gibco), containing 15% Fetal Bovine Serum (FBS, Gibco) and 1% penicillin-streptomycin (100 U/ml-100 µg/mL, Gibco), 10 ng/mL FGF. The cell culture was kept at 37 °C in an atmosphere of 5% CO_2_. Cells were detached from culture flasks by trypsinization, centrifuged and re-suspended. Cell number and viability were assessed with the trypan-blue dye exclusion test.

Each sample (diameter 8 mm, height 4 mm), sterilized by autoclave prior to use, were pre-soaked in culture medium for 72 h at 37 °C.

The samples, used for cell viability and cell morphology at day 7, were seeded by carefully dropping 30 µl of cell suspension (5.0 × 10^4^ cells) onto the scaffold upper surface, and allowing cell attachment for 20 min in the incubator, before the addition of 1 ml of osteogenic culture medium (αMEM Glutamax, 10% FBS, 1% penicillin-streptomycin 100 U/ml-100 µg/mL, 10 mM β-glycerophosphate, 50 µg/mL ascorbic acid, 100 nM dexamethasone).

The U-CUP perfusion bioreactor system (Cellec Biotek AG) was used for the osteogenic gene expression profiling and for cell morphology at day 28. Briefly hADSCs were seeded at 2.0 × 10^6^ cells/scaffold with a bidirectional flow rate of 3 ml/min for 18 h, then all the media were collected and the seeding efficiency was evaluated by counting the cells number left in the culture media after trypan-blue staining.

The cell-seeded constructs were then cultured with a bidirectional perfusion flow rate of 0.3 ml/min for additional 28 days [38]. The osteogenic medium was changed twice a week. All the cell-handling procedures were performed in a sterile laminar flow hood. All cell-culture incubation steps were performed at 37 °C with 5% CO_2_.

### Cell viability assay

Live/Dead assay kit (Invitrogen) was performed according to manufacturer’s instructions. Briefly, the samples were washed with PBS 1x for 5 min and incubated with Calceinacetoxymethyl (Calcein AM) 2 µM plus Ethidium homodimer-1 (EthD-1) 4 µM for 15 min at 37 °C in the dark, the samples were rinsed in PBS 1x [39]. Images were acquired by an inverted Nikon Ti-E fluorescence microscope (Nikon). One sample per group was analysed at day 7.

### Cell morphology evaluation

One sample per group was used for fluorescence (day 7 and 28) and for SEM analysis (day 7 of cell culture). In order to visualize actin filaments, samples were washed with PBS 1x for 5 min, fixed with 4% (w/v) paraformaldehyde for 15 min and washed with PBS 1x for 5 min. Permeabilization was performed with PBS 1x with 0.1% (v/v) Triton X-100 for 5 min. FITC-conjugated Phalloidin (Invitrogen) 38 nM in PBS 1x was added for 20 min at room temperature in the dark [40]. Cells were washed with PBS 1x for 5 min and incubated with nuclear stain DAPI (Invitrogen) 300 nM in PBS 1x for 5 min. The nuclear morphological changes were also evaluated. Images were acquired by an Inverted Ti-E fluorescence microscope (Nikon).

For SEM analysis, after 7 day one sample per group was washed with 0.1 M sodium cacodylate buffer pH 7.4 and fixed in 2.5% glutaraldehyde in 0.1 M sodium cacodylate buffer pH 7.4 for 2 h at 4 °C, washed in 0.1 M sodium cacodylate buffer pH 7.4 and dehydrated in a graded series of ethanol for 10 min each. Dehydrated samples were sputter-coated with gold and observed using Quanta Scanning Electron Microscope (ESEM Quanta 200, FEI).

### Quantitative real-time polymerase chain reaction (q-PCR)

At day 28, cells grown on the β-TCP and HA samples, used as calibrator, were homogenized and total RNA extraction was performed by use of the Tri Reagent, followed by the Direct-zol™ RNA MiniPrep kit (Euroclone) kit according to manufacturer’s instructions. RNA integrity was analysed by native agarose gel electrophoresis and quantification performed by the Qubit® 2.0 Fluorometer together with the Qubit® RNA BR assay kit, following manufacturer’s instructions. Total RNA (500 ng) was reverse transcribed to cDNA using the High-Capacity cDNA Reverse Transcription Kit, according to manufacturer’s instructions. Quantification of gene expression, using Taqman assays (Applied Biosystems), for Alkaline phosphatase (ALP, HS01029144_m1), Osteonectin (SPARC, HS00234160_m1), Osteocalcin (BGLAP, HS01587814_g1), Osteopontin (SPP1, HS00959010_m1), Collagen 15 (COL15A1, HS00266332 m1) and glyceraldehyde 3-phosphate dehydrogenase, used as housekeeping gene, (GAPDH, Hs99999905_m1) was performed by use of the StepOne™ Real-Time PCR System (Applied Biosystems). N. 2 scaffolds for each sample were analysed, using three technical replicates for each experiment. Data were collected using the OneStep Software (v.2.2.2) and relative quantification was performed using the comparative threshold (Ct) method (ΔΔCt), where relative gene expression level equals 2^−ΔΔCt^ [41]. Two scaffolds per group were analyzed in three technical replicates; error bars reflect one standard error of the mean of 3 technical replicates as described elsewhere [42, 43].

### Statistical analysis

Results were expressed as Mean ± SEM plotted on graph. Statistical analysis was made by two-way ANOVA analysis of variance by the GraphPad Prism software (version 6.0), with statistical significance set at *p* ≤ 0.05.

## Results

### Bioceramic scaffolds structure and characteristics

**Fig. 1:**
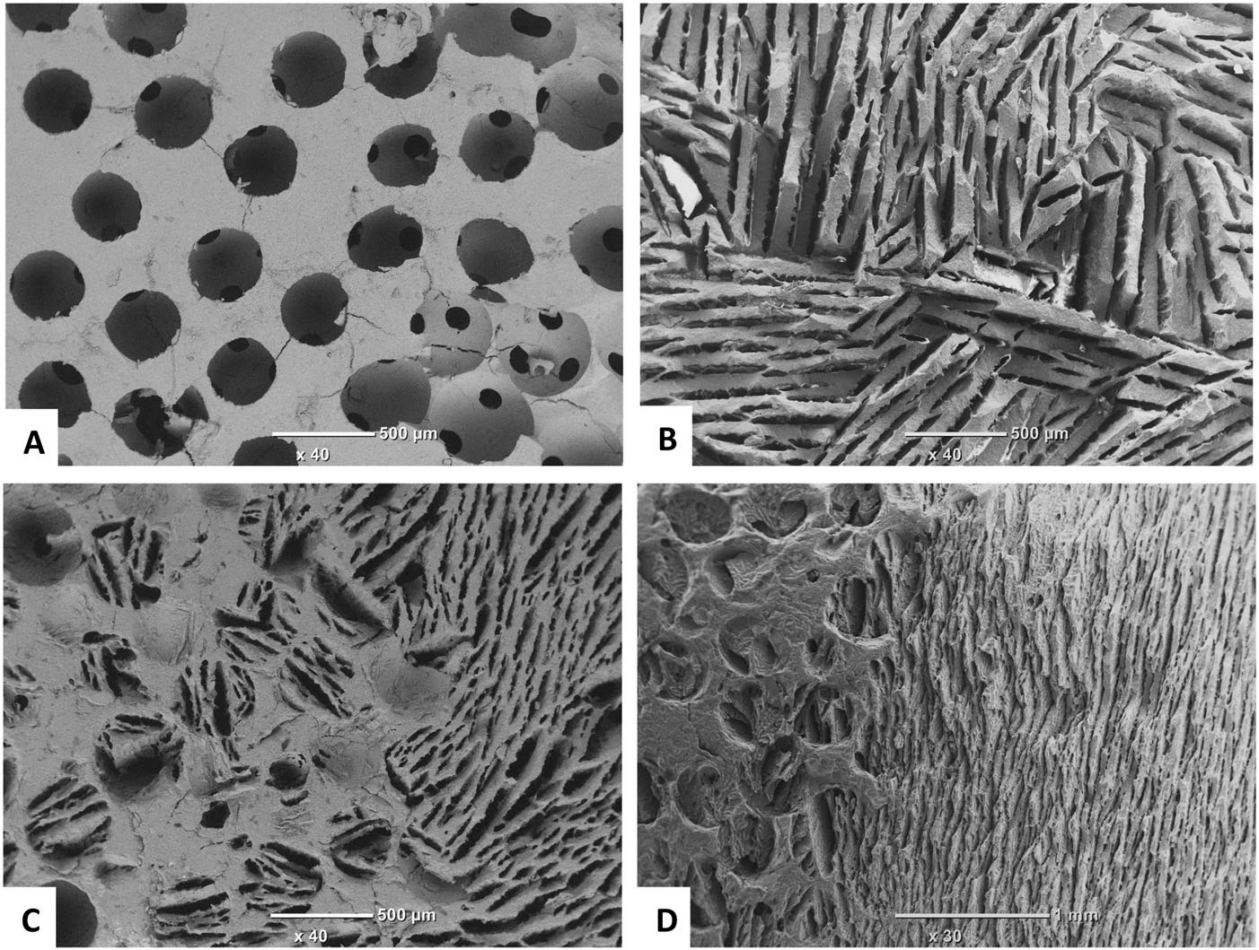
SEM micrographs of ceramic scaffolds: (**A**) HA SS, (**B**) β-TCP CS, (**C**) HA DC shell scaffold in the transversal section and (**D**) cross section of β-TCP DC shell scaffold

**Fig. 2:**
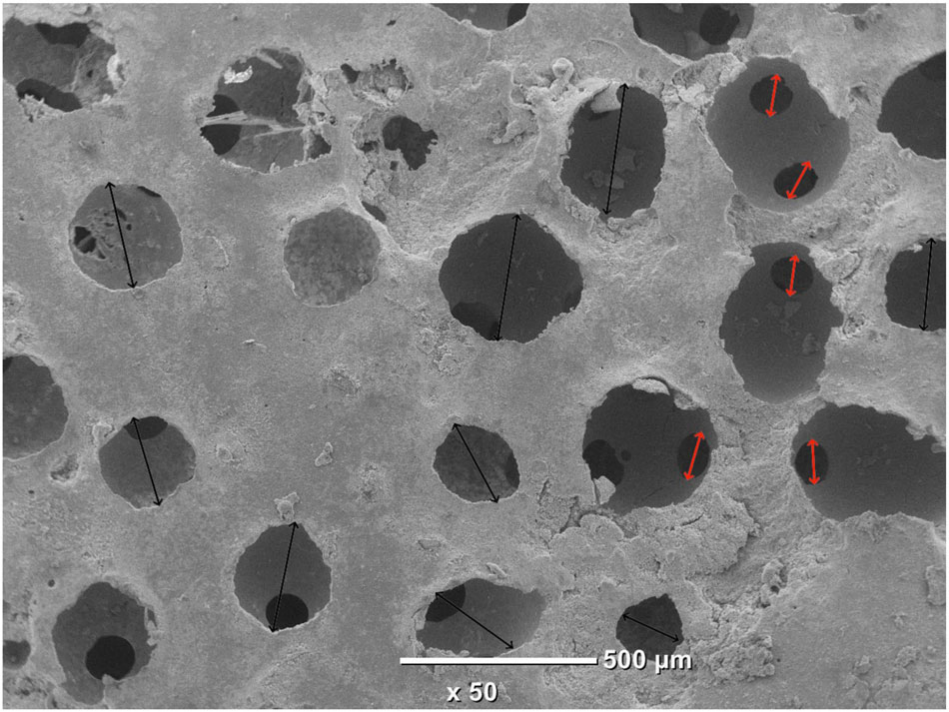
Illustration of the pore and interconnection size measurement procedure for SS samples. Black arrows correspond to pore diameter and red ones to interconnection size

In this study, three types of scaffold structure have been developed from two materials (HA and β-TCP) as shown in Fig. 1. Specimens processed as SS present spherical pore morphology with an interconnection between the pores as observed in Fig. 1A. The pore size of SS has been evaluated by SEM micrograph analysis at a value of about 350 ± 50 µm with an interconnection of 105 ± 15 µm, for both HA and β-TCP. The pore and interconnection size measurement procedure is illustrated in Fig. 2.

**Fig. 3:**
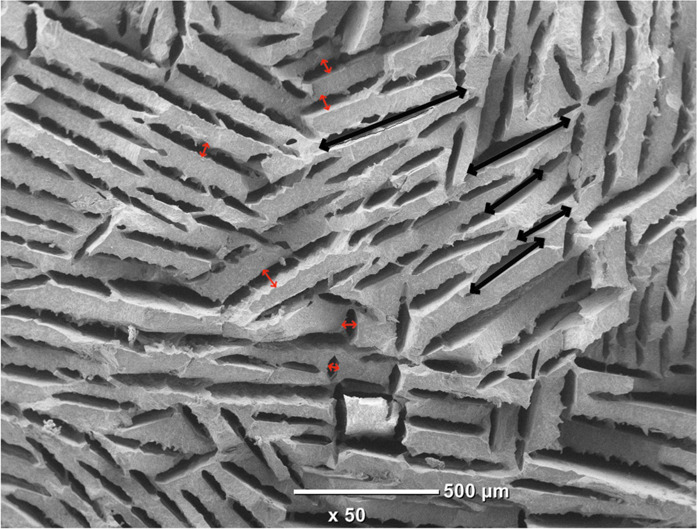
Illustration of the pore size measurement procedure for CS samples. Black and red arrows correspond to long and short axis of the elliptical pore respectively

The freeze casting technique was used for processing the second type of scaffold (CS). This technique yields an anisotropic and elongated pore structure parallel to the freezing direction (lamellar structure) and elliptically shaped pores perpendicularly to the freezing direction, as shown Fig. 1B. The pore size of CS has been evaluated of ~300 ± 150 µm and 45 ± 10 µm for the long and short axis of the elliptical pore respectively. The pore size measurement procedure is illustrated in Fig. 3.

**Fig. 4:**
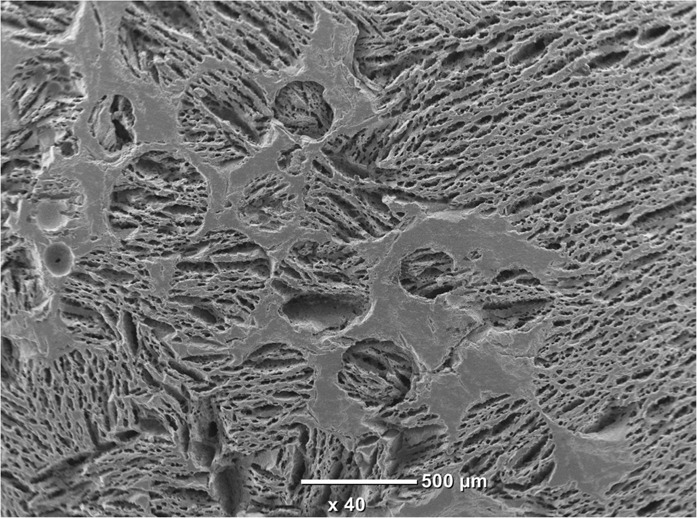
Focus on the interface between the two structures showing the slurry intrusion into SS scaffold during the freeze casting process

The third type of scaffold is a hybrid of both previous scaffold types resulting in the dual core shell structure (DC). The macrostructure is characterised by a spherical pore structure surrounded by the anisotropic lamellar structure. The pore sizes are also of the same order as those of the previous (SS and CS) scaffolds. As can be seen in Fig. 1C-D, the lamellar structure from freeze casting is retained without being disturbed by the presence of the pre-sintered CS. However, at the boundary between the SS and CS region, the CS structure is observed inside the SS part (Fig. 4). This phenomenon could be explained by slurry intrusion during the freeze casting process.

Table 1 summarizes the results obtained for the porosity measurements of all HA and β-TCP specimens. As previously highlighted, the ratio of SS and CS for processing DC specimens has been selected at 1:1 in order to have the same volume of each structure. However, as a significant amount of slurry flows inside the SS structure during freeze casting, the ratio change as it can be observed with the porosity level values of DC specimen. Indeed, the porosity level of DC should be around 50%, considering the 1:1 ratio, however the value is lower of about 42%. By assuming a similar shrinkage for both structures, volume of CS is thus larger than SS volume. This phenomenon (slurry inside spherical pores) was not observed in a previous study [33], as only the boundary was slightly penetrated by the slurry. This could be as a result of the reduction of the sample size in the present work, the samples being smaller in order to be suitable for the in vitro biological tests. Indeed, if the ratio between SS and CS is kept constant and with the decrease of size, the penetration is almost similar in term of depth of penetration but not in respect to the overall volume, a size is reduced.

**Table 1:** Porosity level of HA and β-TCP scaffolds

	Porosity (%)		
	Spongy scaffold	Cortical scaffold	Dual core shell
HA	63.1 ± 1.3	37.9 ± 2.1	41.7 ± 3.6
TCP	63.7 ± 1.0	37.4 ± 1.1	41.6 ± 1.8

**Fig. 5:**
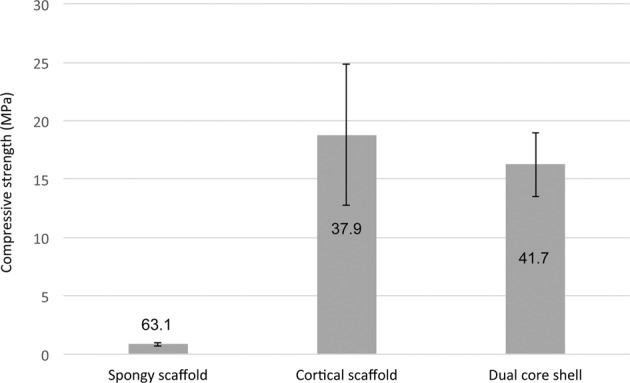
Compressive strength of ceramic scaffolds as a function of the macrostructure (Tag values are the porosity level)

In addition to the morphological characterisation, compressive strength of the HA scaffolds was determined as a function of structure. Mechanical results have been plotted in Fig. 5. No preferential rupture mode was observed whatever the structure with all samples exhibiting classical brittle failure.

### 3D Stem cell culture viability

**Fig. 6:**
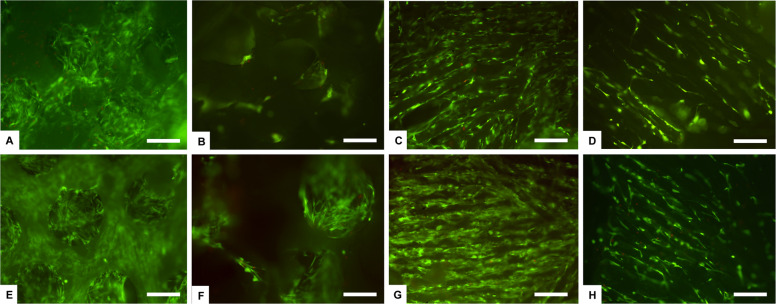
Cell viability analysed by the Live/Dead assay at day 7. Calcein stains live cells in green, Ethidium homodimer-1 stains dead cells in red. **A**–**D** HA samples and (**E**–**H**) β-TCP samples. In detail: (**A**) HA SS upper surface; (**B**) HA SS inner surface. **C** HA CS upper surface; (**D**) HA CS inner surface. **E** β-TCP SS upper surface; (**F**) β-TCP SS inner surface. **G** β-TCP CS upper surface; (**H**) β-TCP CS inner surface. Scale bars 200 µm

For the in vitro assessment, human adipose tissue-derived stem cells (hADSCs) were used. First, the hADSCs were cultured directly in contact with the HA and β-TCP with both macrostructural porosity types CS and SS. After 7 days of culture, the qualitative Live/Dead assay showed a very high ratio of viable cells without any differences among the groups (Fig. 6). These data confirmed that bioceramics represent a very biocompatible material [44, 45]. Even if the samples have different porosities, hADSCs were able to colonize the inner part of the scaffolds showing a very good viability (Fig. 6B, F, D, H).

### Stem cells morphology and scaffolds colonization

**Fig. 7:**
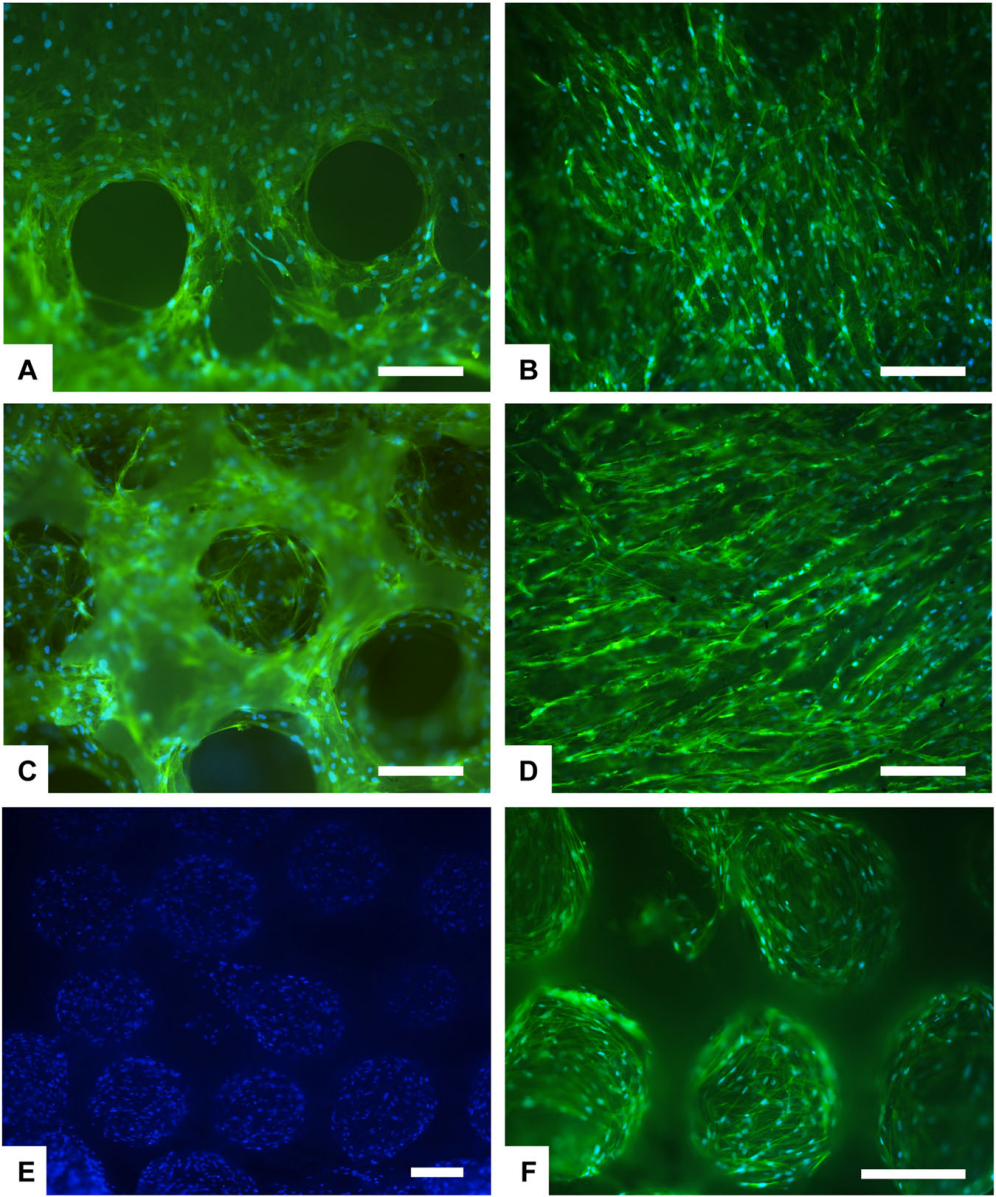
Analysis of cell morphology and scaffold colonization by phalloidin staining at day 7 (**A**–**D**) and at day 28 in bioreactor (**E**, **F**). Phalloidin in green stains for actin filaments and DAPI in blue stains for cell nuclei. **A** HA SS; (**B**) HA CS. **C** β-TCP SS; (**D**) β-TCP CS. **E**, **F** β-TCP SS. Scale bars 200 μm

**Fig. 8:**
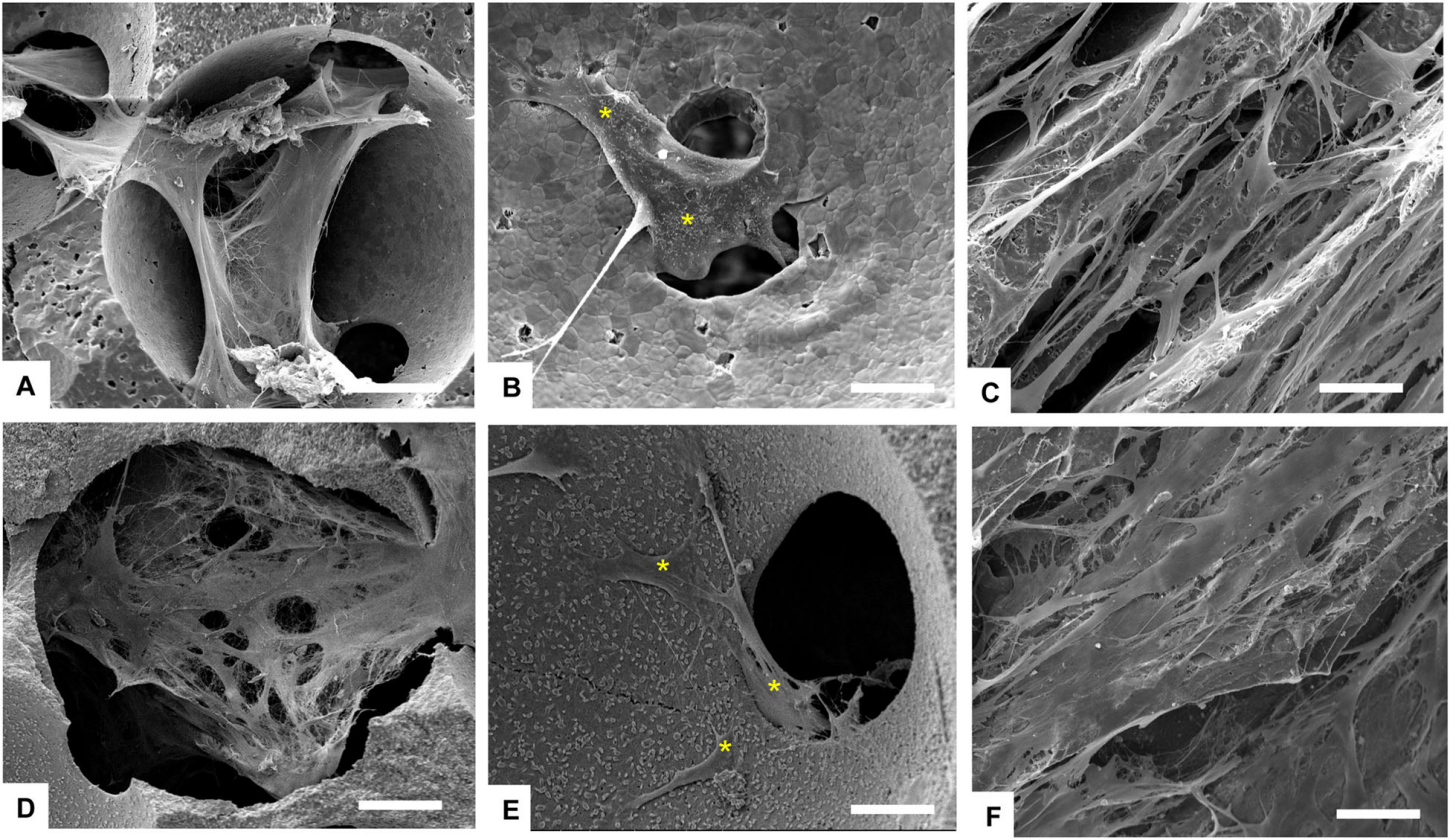
SEM analysis of cells grown on ceramic scaffolds after 7 days. Yellow asterisks indicate some cells. **A**, **B** HA SS; **(C**) HA CS; (**D**, **E**) β-TCP SS; (**F**) β-TCP CS. Scale bars: (**A**, **D**) 100 µm; (**B**, **C**, **E**, **F**) 50 µm

The ability of cells to adhere to a biomaterial surface is an early effect of the regeneration process [46, 47]. Cell surface interaction and cell adhesion are complex processes involving the reorganization of cytoskeleton proteins like actin [48]. Phalloidin stains actin filaments and the image Fig. 7 show attached cells exhibiting their characteristic spindle shape, confirmed also by scanning electron microscopy (Fig. 8).

In both HA SS and β-TCP SS samples, the random and highly interconnected porosity obtained by the replica technique, is suitable for a rapid cell migration inside the scaffold (Figs. 7 and 8). hADSCs can easily bridge the pores and have established steady cell-cell and cell-material interactions. After 28 days of culture in bioreactor, the inner part of random pore scaffolds (HA SS, β-TCP SS) is fully covered by intricated cell-cell connections (Fig. 7E, F). In the aligned porosity samples, obtained by freeze casting technique (HA CS, β-TCP CS), cells were seen to adhere preferably following the aligned structured. However, after 7 days the upper surface is completely covered by a cell layer, and some cellular extensions stretched towards the inner scaffold surface are visible (Fig. 8C, F).

### Osteogenic response in long-term bioreactor test

**Fig. 9:**
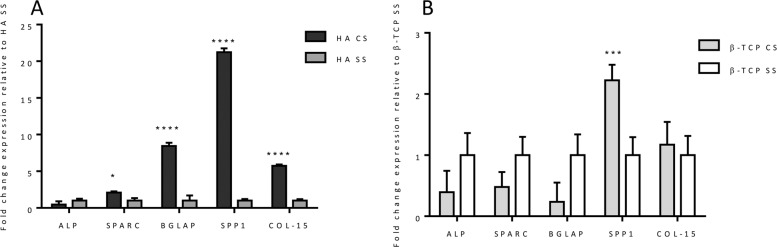
Relative quantification (2-ΔΔCt) of ALP, SPARC, BGLAP, SPP1 and COL15A1 expression as markers of osteogenesis differentiation for hADSCs, after 28 days of culture in bioreactor. The graphs report the mean and standard error of the samples with respect to the expression of control (**A**, HA SS; **B** β-TCP SS) (*p ≤ 0.05; ***p ≤ 0.001; ****p ≤ 0.0001)

The U-Cup perfusion bioreactor was used to better conduct a 4-week experiment to investigate in vitro cell-scaffold interactions in term of gene expression profiling. In this study, two different data analysis have been performed in order to demonstrate the specific effect of chemical and morphological features of ceramic scaffolds, designed for bone regeneration, on cell behaviour. The results showed that the CS porosity is extremely inductive of the expression on the late stage genes involved in the machinery of osteogenic differentiation (Fig. 9). Particular relevance assumes the gene expression profile observed in HA CS compared to HA SS, used in this analysis as control (Fig. 9A). The significant over-expression of BGLAP, SPP1, COL-15 (p value ≤0.0001) and SPARC (p value ≤0.05), indicated the not only the AdMSCs have started the ostegenic differentiation process, but also, after 28 days of dynamic culture, these cells upregulated the genes typically expressed in the active mature osteoblasts [49–52].

Although less marked, the same trend has been showed in the cells cultured on β-TCP CS when compared to the biological effect of β-TCP SS (Fig. 9B). β-TCP CS significantly upregulated the expression of SPP1 (p value ≤0.001), encoding osteopontin, a fundamental protein involved in the cell interaction and in the anchoring to the extracellular matrix [53].

**Fig. 10:**
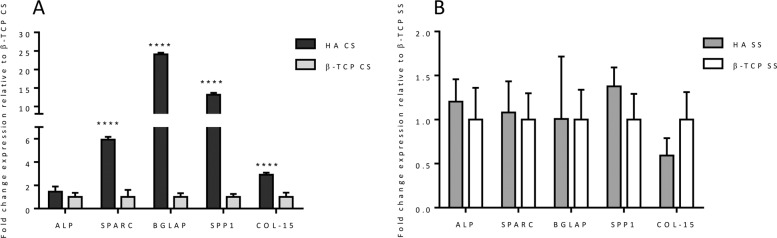
Relative quantification (2-ΔΔCt) of ALP, SPARC, BGLAP, SPP1 and COL15A1 expression as markers of osteogenesis differentiation for hADSCs, after 28 days of culture in bioreactor. The graphs report the mean and standard error of the samples with respect to the expression of control (**A**, β-TCP CS; **B**, β-TCP SS) (****p ≤ 0.0001)

Moreover, in order to assess the biological effect of the chemical composition, a comparison between the two different materials with the same porosity has been performed (Fig. 10). The results showed a significant upregulation of the osteogenic related genes (SPARC, BGLAP, SPP1 and COL-15 *p* value ≤ 0.0001) exerted by HA CS compared to β-TCP CS (Fig. 10A). On the contrary HA SS did not induce any kind of bioactivity on hADMSCs if compared with β-TCP SS (Fig. 10B).

### Dual core shell structure ceramic scaffolds: preliminary data

**Fig. 11:**
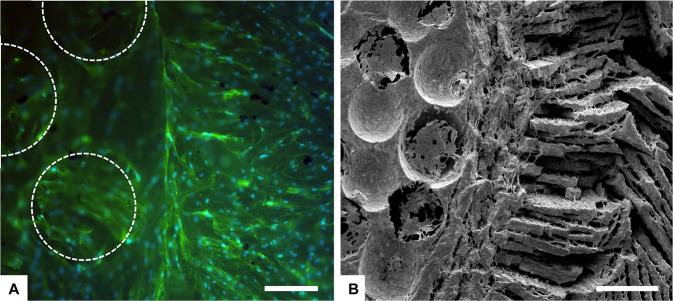
Cell distribution analysis performed by Actin staining (phalloidin in green stains for actin filaments and DAPI in blue stains for cell nuclei), (**A**) and SEM analysis, (**B**) on β-TCP dual core shell structure scaffold at day 7. Dashed lines show pores of the internal part of the dual scaffold. Scale bars: (**A**) 200 µm; (**B**) 500 µm

The dual core shell scaffolds include in one-bone scaffold solution the different porosity of the cortical and cancellous bone. After the deep analysis performed in static and dynamic condition in bioreactors up to 28 days, a preliminary study was performed on the dual core shell structure scaffolds. hADSCs were cultured directly in contact with the samples and we analysed the cell morphology and distribution after 7 days. The results showed a uniform distribution of the cells, without any differences among the groups, that follow the behaviour already seen in the previous analysis where the HA and β-TCP scaffold structured were analysed separately (Fig. 11). The dual core shell scaffolds represent a very promising design for bone regeneration resembling the two different bone architectures.

## Discussion

Biomaterial science increasingly seeks biomimetic scaffolds that truly reproduce multiple structural characteristics of bone tissue with the final aim to regenerate critical bone defects. Of all the biomaterials, bioceramics still represent the best biomimetic materials reproducing the chemistry of the organic bone phase and able to communicate to the cells accurate signals promoting bone regeneration [41–43]. Moreover a highly interconnected porous structure (typically a pore content above 50 vol.%) is essential to enable full integration of the scaffold once it is implanted. In this study HA and β-TCP bioceramic scaffolds with different bioinspired porosity, mimicking the spongy and cortical bone tissue, were studied. The scaffold macroscopic organisation has previously been shown to have an important impact on cell proliferation [54]. The scaffold porosity analysis showed that powder characteristics have a slight impact on the porosity of the sintered structures, and for both processing routes, the overall porosity is controlled by the PMMA bead structure and the water content, for SS and CS respectively. As it could be expected, the impact of porosity level is important in mechanical performance: the higher the porosity is, the lower the compressive strength will be. However, the macrostructure should be taken into account because the anisotropic part of DC is oriented thus improving the compressive strength along this direction [55]. The chemical composition almost identical to that of the mineral phase of bone and the bioinspired porosity confer to these scaffolds an excellent biomimetic property that was analysed in vitro looking at hADSCs behaviour once seeded in direct contact with the specimens. hADSCs were used since they are a very promising source of autologous stem cells that could certainly be employed in tissue engineering to boost the bone regeneration [56]. Moreover hADSCs could be easily obtained and expanded in vitro from adipose tissue by minimal invasive surgery (i.e., liposuction) [57]. The excellent cells viability and cell adhesion to the biomaterials highlighted the biomimicry of the scaffolds. In fact the attachment phase of cell adhesion occurs rapidly and involves physicochemical linkages between cells and material. Cell spreading is an essential function of a cell, which has adhered to a surface and precedes the function of cell proliferation, to provide a cell-covered surface, and cell differentiation [46, 47]. In addition the interconnected pore network is essential to obtain an homogenous scaffold cell colonization and enhance the tissue regeneration process [58]. To deeply investigate the bioactivity of the proposed scaffolds, an additional long term in vitro study was carried out in bioreactor in presence of hADSCs. The direct perfusion permits a uniform hADSCs seeding, and mimics the physiological conditions in term of interstitial fluid flow and associated induced shears [59–62]. The osteogenic response was analysed in order to evaluate specific effect of both chemical and morphological features of ceramic scaffolds. The results support the evidence that biomaterial chemistry and architecture deeply influences the cell fate especially at prolonged time culture. It is possible to assert that the HA composition and the presence of organized and hierarchical architecture of the CS scaffold acts in synergy as a potent activator of the molecular pathway involved in the osteogenic differentiation. A preliminary test was done on dual core shell ceramic scaffolds that summarized in one-bone scaffold solution the diverse porosities, and the results confirmed the data obtained on the single scaffold structure. The multi-scale structure of these DS scaffolds confers unique features fundamental for long bone tissue regeneration.

## Conclusion

The specific chemical-physical features of these multi morphology scaffolds may guide cellular behaviour, with specific effect on the expression on late stage osteogenic genes. The similarity of the HA and β-TCP, in term of chemical composition, to the native bone has been confirmed as a key factor in biomaterials for bone tissue regeneration. Moreover, the cortical bone bioinspired porosity strongly enhanced osteogenic differentiation of mesenchymal stem cells compared to the spongy bone porosity. Considering the results, the dual core shell design of bioceramic materials represent a promising one-scaffold solution for critical bone defect where physiological multi-scale structures are required. The cortical structure boosting the osteogenic process could give, upon implantation, an initial functional biomechanical support in a quite short time due to a rapid new bone regeneration, that will be followed by subsequent bone maturation in the inner spongy structure.
